# “Antibiotics Can Work as a Contraceptive:” Contraceptive Knowledge and Use Among University Students in Calabar, Nigeria

**DOI:** 10.3389/frph.2021.665653

**Published:** 2021-07-07

**Authors:** Ibitola Asaolu, Nidal Kram, Christopher Ajala, Ememobong Aquaisua, Akinsola Asaolu, Kylie Kato-Lagumbay, Alice Abuh, Moses Bernand, John Ehiri

**Affiliations:** ^1^Mel and Enid Zuckerman College of Public Health, University of Arizona, Tucson, AZ, United States; ^2^Center for AIDS Intervention Research, Medical College of Wisconsin, Milwaukee, WI, United States; ^3^Cross River Health and Demographic Surveillance System, University of Calabar, Calabar, Nigeria; ^4^Faculty of Public Health, College of Medicine, University of Ibadan, Ibadan, Nigeria

**Keywords:** contraceptive, Nigeria, youth, university student (MeSH), qualitative study, contraceptive methods, family planning, contraceptive practices

## Abstract

**Introduction:** Many sexually active youths who wish to delay pregnancy are not using any form of modern contraceptives. In sub-Saharan Africa, less than 1 in 5 sexually active youth do not use contraceptives. In Nigeria, 48.4% of all sexually active unmarried women have an unmet need for contraception. Although the literature is replete with information on structural barriers to modern contraceptives, there is limited scholarship on contextual factors that may inhibit modern contraceptive use among Nigerian youth. This study uses a qualitative research approach to assess knowledge and use of natural, modern, and folkloric contraceptive methods among a sample of university students in Calabar, Nigeria.

**Methods:** This study used data from focus group discussions among women and men in university halls of residence, all of whom were unmarried. Also, three male pharmacists and three female community health workers were interviewed. All focus group discussions and interviews took place in August 2017 and were conducted in Calabar Metropolis, Cross River State, Nigeria. The audio recordings were transcribed into detailed summaries of the interviews and focus group discussions. All data analysis was completed using Atlas.Ti (version 8).

**Results:** University men and women have limited knowledge of and application of natural and modern contraception. Participants listed folkloric methods of contraceptives, including repurposing pharmaceuticals (e.g., antibiotics, quinine, and Andrews Livers Salt-a laxative) as contraceptive agents. Respondents also discussed the use of non-pharmaceuticals such as water, salt solution, and squatting after intercourse as contraceptives. Generally, university students defaulted to withdrawal, calendar method, and emergency contraceptives as preferred methods of contraception. Lastly, condoms were used among participants in causal sexual encounters. In dating relationships, however, both male and female students cited their partners' hesitancy to condom use as such act could indicate distrust.

**Conclusion:** Awareness and use of modern contraceptives are limited among university students in Calabar, Nigeria. The use of folkloric contraceptives could lead to bodily harm and unintended pregnancy. Further research is needed to elucidate factors that promote use of folkloric methods of contraceptives. Integration of contraceptive awareness into health promotion services for young people may help to dispel myths about folkloric methods of contraceptives while promoting awareness and use of safe and effective contraception.

## Introduction

Contraceptive use is pivotal to reducing maternal and infant mortality (1), decreasing child marriage and unintended teen births, and supporting economic development (2). Despite global efforts to improve access to, and use of, contraceptive methods, the unmet need for contraceptives across the world remains high. The latest figures on contraceptive use reveal that 190 million women of reproductive age (15–49 years old) have an unmet need for contraception (3). Sub-Saharan Africa bears the highest burden for the unmet need for contraception, with an estimated 1 in 4 women of reproductive age having an unmet need for contraception (4).

Nigeria, through the Family Planning 2020 Initiative, set a goal to increase the modern contraceptive prevalence of all women from 16% in 2017 to 27% in 2020 (5). However, the contraceptive prevalence for married women, using both traditional and modern methods, remains low at 14% (6). In Nigeria, 18.9% of currently married and 48.4% of all sexually active unmarried women have an unmet need for contraception (6). To promote the health and welfare of Nigeria's women and infants, it is imperative to further understand the factors—such as lack of knowledge, limited use of highly effective contraceptive, and marital status—that contribute to the low uptake of modern contraceptives.

First, the low contraceptive prevalence among men and women in Nigeria may be the result of limited knowledge of contraceptive methods, which varies by the type of contraceptive and marital status (7). The most common forms of contraceptives identified by women in Nigeria are pills or injectables (82%) and male condoms (77%) while standard days method (23%) and male sterilization (17%) were least identified (6). Interestingly, sexually active unmarried women are more knowledgeable about female condoms and emergency contraceptives (73 and 66%, respectively) than married women (42 and 37%, respectively) (6). Although there is limited scholarship on contraceptive use among men in Nigerian, majority (70%) of sexually active men do not use any form of contraceptives (8). Among men in Nigeria who used contraceptives, condoms were the predominant form used (20%), followed by withdrawal (3.1%), and periodic abstinence (2.5%) (8). The gap in knowledge and use of contraceptive methods in Nigeria reveals the need for targeted interventions that promote contraceptive use among those who wish to delay or prevent pregnancy.

Second, contraceptive use among women in Nigeria differs by the type of contraceptive and woman's socioeconomic factors. About 1 in 10 (10.5%) women in Nigeria use a modern contraceptive while 3.8% used a traditional contraceptive (6). Women with formal education are more likely to use modern contraceptive methods than those with no formal education; the use of modern contraceptive methods is higher among married women with more than secondary education (23%) compared to those with no education (4%) (9). Similarly, married women in the highest wealth quintile (22%) have a higher prevalence of modern contraceptive use than women in the lowest quintile (4%) (6).

Third, the type of contraceptives used also varies by marital status. Modern contraceptive use among sexually active unmarried women in Nigeria (28%) is higher than among currently married women (12%) (6). This finding reaffirms the low contraceptive uptake among married women, as only 13% of married women used modern contraceptive methods (9). Specifically, unmarried women have a higher proportion of male condom use than married women (18.5 vs. 1.6%, respectively) (9). Additionally, research has documented the misuse of pharmaceuticals (e.g., analgesics) as contraceptives among unmarried university women (10). On the contrary, married women in Nigeria had a higher prevalence of using IUDs (0.8 vs. 0.2%) and contraceptive implants (3.4 vs. 1.4%, respectively) than unmarried women (6). While acknowledging that fertility intent informs the choice of contraceptives, unmarried women tend to use less-effective contraceptives and sometimes misuse pharmaceuticals as contraceptives. Therefore, it is important to promote awareness of, and access to, safe and reliable contraceptives that will suit young people's fertility preferences.

Given the limited knowledge, high unmet need for contraceptive, and low prevalence of modern contraceptive use in Nigeria, this qualitative study was conducted to elucidate factors that contribute to the low use of modern contraceptives. The study examined knowledge of contraception and the various forms of contraceptive methods—natural, traditional, modern, and folkloric—used among university women and men in Calabar, Nigeria.

## Methods

### Study Context

This study was conducted as part of a larger qualitative research project consisting of focus group discussions (FGDs) among postnatal women at primary healthcare centers, university students (males and females) at their halls of residence, and in-depth interviews with community health workers and pharmacists. All FGDs and interviews took place in August 2017 and were conducted in Calabar Metropolis, Cross River State Nigeria. Calabar Metropolis consists of two Municipal Councils: Calabar South and Calabar Municipality. Calabar Metropolis—the capital of Cross River State, is largely urban although it has some rural/semi-urban settlements. The predominant religion practiced in Calabar Metropolis is Christianity (11, 12).

Given the differences in demographic characteristics and reproductive health needs of university-based (i.e., unmarried) participants and clinical-based postpartum (i.e., mostly married) women, the current report was limited to university women and men. University students were recruited from the two major public universities in the city: Cross River University of Technology and University of Calabar. These two universities serve a student population drawn from various states and regions in Nigeria. The University of Calabar was founded in 1975; it has a graduate school, 10 faculties, three Institutes, and student population of 40,645 (13). Cross River University of Technology was established in 2002; it has eight faculties spread across four cities in Cross River State (14).

### Ethics Approval

This research study was approved by the Health Research Ethics Committee of the University of Calabar Teaching Hospital, Calabar, Nigeria under the protocol number UCTH/HREC/33/562 and the Cross River State Health Research Ethics Committee of the State Ministry of Health. All participants signed an informed consent form addressing the risk and benefits of participation. To protect their identity, each participant provided a preferred alias, by which they were addressed during the focus group discussion. Students were compensated for their time (N500, approximately US $2 in August 2017).

### Data Collection, Transcription, and Analysis

Four FGDs and six interviews were conducted with university students, community health workers, and pharmacists. Three focus groups were conducted with 17 purposively sampled undergraduate women, and one focus group with eight snowball sampled undergraduate men. Of the six interviews conducted, three were with pharmacists and three with community health workers. Semi structured FGD questions and interview guides were developed based on questions from Ochako et al. (15), Mugisha and Reynolds (16), and Family Health International (17). Briefly, university students were asked about: (1) their knowledge of ways to prevent unintended pregnancy; (2) different methods of contraceptives; (3) beliefs about pros and cons of various contraceptive methods; (4) source of contraceptives; (5) cost of purchasing contraceptives; (6) forms of contraceptives they used; (7) factors that inhibited or facilitated their use of contraceptives.

All focus groups and interviews were conducted in English, audio-recorded, and transcribed into detailed summaries. IA, a female researcher experienced in qualitative data collection, facilitated interviews and FGD sessions among university women, with AAb as the note taker. MB, an experienced undergraduate male student, led the FGD among undergraduate male students, with IA as the notetaker. Focus groups ranged between 45 and 96 min. IA conducted all the interviews, and they lasted up to 28 min. There were no previously established relationships between participants and members of the research team. Data collection stopped after saturation was reached.

The audio recordings were transcribed into detailed summaries of the interviews and focus group discussions. The theory of planned behavior (TpB) (18) provided structure for interpreting data from the FGDs and interviews. According to the theory, human behavior is guided by three kinds of considerations: beliefs about the likely outcomes of the behavior and the evaluations of these outcomes, (behavioral beliefs); beliefs about normative expectations of others and motivation to comply with these expectations (normative beliefs), and beliefs about the presence of factors that may facilitate or impede performance of the behavior, and the perceived power of these factors (control beliefs). IA, KK, and NK reviewed the guides, data from focus groups, and interviews and then developed a codebook comprising deductive and inductive codes. Deductive codes were based on key issues addressed in interviews while inductive codes were based on emergent themes. All data analysis was completed by IA and KK using Atlas.Ti (version 8). Data were independently coded and then discussed to address inconsistencies or disagreements in coding.

## Results

The study used data from FGD with 17 female and eight male undergraduate students. The female undergraduate students were on average 22-years old, unmarried with no children, and mostly (*n* = 12; 70%) in a romantic relationship. The average age of undergraduate males was also 22-years; all the men were unmarried while half of them (*n* = 4) were in a romantic relationship. In line with constructs of the TpB (18), the results describe students' knowledge of contraception, methods of contraceptives, source of information on contraceptives, and forms of contraceptives they used.

### Methods of Contraceptives Used

FGD participants talked about different methods of contraceptives. Many of them mentioned methods of contraceptives that are different from the known natural or modern contraceptives.

### Repurposing Pharmaceuticals

Participants mentioned repurposing pharmaceuticals (e.g., laxatives and antibiotics) as contraceptives. The students mentioned three types of pharmaceuticals (i.e., laxatives, antimalarial, and antibiotic) that served as contraceptives.

Andrews Liver Salt: laxative

*My friend said she takes Andrews Liver Salts before having sex. She puts it inside water and drinks it. Because once you drink it and you have sex and the man ejaculates, the sperm can no longer function because the Andrews Liver Salt you drank will make the sperm weak*. (Female FGD)

Quinine: anti-malarial

*I have a nurse friend who is married. She used to tell me that the moment she had sex, she would take white Quinine although it made her drowsy. She would take one tablet immediately after she had sex*. (Male FGD)

Ampiclox: antibiotic.

*You go (to a pharmacy) and say I want Ampiclox. Some people take it for infections, but it can also work as a contraceptive. Most of the people (pharmacists/pharmacy technicians) will know what you want to use it for* (Female FGD)

Many university students agreed on the efficacy of ampiclox in preventing unintended pregnancy.

Vitamin C

*I've learnt of vitamin C too. Before your period just take two tablets of white vitamin C, it will make your canal acidic. So, when he (a man) comes on you, it (acidic canal) will definitely kill the sperm*. (Female FGD)

### Pharmacists' Perspective on Repurposing Medications as Contraceptives

The tendency for women to repurpose pharmaceuticals was identified by a pharmacist and exemplified by participants.

*Sometimes, some of them buy drugs that they're using for the secondary properties, the adverse effect*. (Pharmacist # 2)

In addition, a community pharmacist described how some women would use certain medications purposefully for their secondary properties because these medications have a side effect of causing miscarriage.

*The younger girls are mostly pregnant already and they say they've been told to take certain medication to terminate the pregnancy…And a few when they take this, it will remove whatever implant or pregnancy of how many months - they don't care. At that particular point they find themselves, they just want to get rid of the pregnancy*. (Pharmacist # 2)

For instance, a young woman in the university focus group mentioned how Misoprostol, a medication for stomach ulcer, could be used for abortion. This participant, however, could not distinguish whether the effect of this medication was to prevent conception or end an existing pregnancy.

*Yes, it (misoprostol) can be used for an abortion. It depends how many months; like the original one is 3 (pills in a pack), but most of them are fake. If you go to some hospitals, it's just three in a pack; they have to cut it (misoprostol) for you to take. The (certified) pharmacies themselves will always demand for a prescription. There are some hard drugs for which prescription is necessary. You can't just sell it unless it's a backyard business (illegally run Pharmacy)*. (Female FGD)

### Non-pharmaceuticals

Students, especially females, mentioned using non-pharmaceuticals as forms of contraceptives. These women described how they have either used or heard of water, salt, and squatting as forms of contraceptives.

Water and salt

*My friend told me that after unprotected sex she would use water and put salt into water… Yes, that's what she said (*that she used water and salt immediately after sex*), and she's a medical student (*thus lending credence to this method*)*. (Female FGD)

*About the water, once you can take about 30 centiliters of water, after a while you see the urine. After a while, you'll see it (semen) in your pants*. (Female FGD)

Squatting

*Just relax, at the end of it* (intercourse)*, once you are up, you just go to the bathroom to squat for 30 minutes try pushing it out as if you are peeing. And you notice that it* (semen) *is coming out on its own without using contraceptives. You squat; when you squat, you push it as if you're trying to push something with force. Stay there* (in the position) *for 30 minutes, the sperm* (semen) *will definitely come out*. (Female FGD)

Also, participants talked about other non-pharmaceuticals (e.g., dry gin, Lipton Tea, and Schweppes) that are used as contraceptives.

### Natural and Modern Contraceptives

Lastly, participants mentioned withdrawal, condoms, postinor (a common emergency contraceptive in Nigeria), and the calendar method as other forms of contraceptives. The two commonest forms of modern contraceptives among participants were condoms and emergency contraceptive pills while calendar and withdrawal methods were the two commonest forms of natural methods.

### Contraceptive Preference

Although participants described various methods of contraception, they highlighted their preferences for withdrawal, safe period (calendar method), and emergency contraceptives rather than condoms. For instance, undergraduate males reported that sometimes they had sex without condoms because their partner might no longer be interested in sex if the men went searching for condoms right before intercourse.

*When the anointing falls (sexual urge), you use the withdrawal method because your partner might no longer be in the mood by the time you return after going out to purchase a condom*. (Male FGD).

In other cases, participants noted that they used safe period rather than condoms.

*Your safe days, the natural method it's just for you to know your menstrual cycle; when you are safe and when you are not safe. So, when the condom is not there, you can easily use your safe period. (*Female FGD*)*

*I don't really fancy condoms.…I tend to go with safe period*. (Male FGD)

Also, healthcare providers highlighted the preferential use of certain contraceptives by their clients. For instance, a pharmacist mentioned how single women preferred to use emergency contraceptives over other forms of modern contraceptives.

*Unmarried ones, adolescents, are not too bothered about contraceptives; they are only looking for these first-line ones to prevent pregnancies. They don't want to have any children just yet… So, they just prefer the immediate ones like the levonorgestrel, the postinor (emergency contraceptives). They take that and they know that they are safe (Pharmacist 1)*

### Condoms

In general, participants (males and females) were reluctant to use condoms. Condom was preferred in the context of casual or transactional sex instead of a committed and monogamous relationship. In some cases, females who did not trust their boyfriends would defer to use condoms until they were both tested for different STIs.

*When my boyfriend was in Port Harcourt, he said he wanted to do it raw (have unprotected sex) and feel the impact. “I told him for this night ‘let's use a condom and tomorrow we can go to the hospital because I'm very careful. We can go to the hospital and do the necessary test and after the result is out, we can do without condoms.” He agreed and said no problem*. (Female FGD)

*I have never used condoms before even though I have been with my boyfriend for about 8 years, and I've not gotten an infection…. But, he's so careful; he uses condoms with every other girl*. (Female FGD)

*I don't really fancy condoms. I use condoms when I accidentally meet someone, and something has to go down (i.e., have intercourse with them). (*Male FGD*)*

Also, respondents reported situations where their romantic partners were hesitant to use condoms. In two instances, undergraduate males reported that their girlfriends took offense to their suggestion of condom use. In each case, each woman asked her boyfriend if he did not trust her or whether she was a sex worker.

*Ninety percent of women I've had sex with are not my girls (i.e., I'm not in a relationship with them). They are cash and carry (sex workers), so at that point in time you tend to use condom. The remaining ten percent, I use safe period. There was an issue with a girlfriend who asked me, ‘why use a condom, am I a prostitute?'. When you know more, you tend to trust that person. In relationships, trust is important*. (Male FGD)

*Personally speaking, when we first started off, condom was not in the picture. We did HIV testing and listened to health education messages. So, I brought up condom use, but my girlfriend was not happy. She asked if I didn't trust her*. (Male FGD).

Lastly, participants disclosed they would not use condoms because their partners believed that condom-less sex was more pleasurable than sex with condom.

*I have a friend she was complaining to me that her boyfriend did not want to use condoms. He wanted to go natural, he claimed that he did not enjoy sex with condom. So, my advice to her was to look for any other means and protect herself because if she gets pregnant, she will be the one to bear the consequences. (*Female FGD*)*

Healthcare providers also echoed the students' sentiment about “natural” sex referenced above as a deterrent to condom use.

*The unmarried ones believe that using condoms, for example, does not make sex pleasurable enough, and they feel their girlfriends should be able to calculate their safe period properly (Pharmacist 1)*

### Contraceptive Knowledge

This sub-theme describes participants' definition of contraception and contraceptive methods. Generally, participants mentioned their awareness of modern contraceptives; they mentioned condoms, injectables, emergency contraceptives, male and female condoms, contraceptive implants, and intra-uterine devices.

### Lack of Awareness on Contraceptive

Participants also described methods of contraceptives that were medically inaccurate (e.g., sexual position) or partially inaccurate (e.g., effectiveness of intra-uterine device).

*Sexual position also matters. When a man lies on his back and the girl is on top, the chances of getting a girl pregnancy are very slim*.

(Male FGD)

*The one I know of, but I have not done it, is the one where they insert a device inside your womb for 12 years and the one where they inject you for 2 months* (Female FGD)

Participants' description of contraceptives also included wrong or incomplete definitions of contraceptive method as evident in their description of calendar days, which they commonly referred to as “safe period.” While some participants knew *safe period* as a means of contraception, others could not accurately describe how to use the calendar method (*safe period*).

*Talking about safe period, the first period which lasts three to four days is very dangerous. You can get pregnant anytime. The second one lasts up to fourteen days, so it is safe*. (Female FGD)

Withdrawal was also commonly mentioned as a form of contraceptive across the focus groups. The efficiency of withdrawal as a form of contraceptive was heavily discussed in the undergraduate male FGD.

*I did Biology in school. The precum does not get you pregnant, it cleans your urethra and prevents STI. When the real stuff comes, when you ejaculate that's what gets the woman pregnant…So, it depends on how experienced you are in practicing the withdrawal method*. (Male FGD).

Furthermore, the limited or lack of knowledge of modern contraceptives came up as a deterrent to use. For instance, undergraduate males were unaware of intrauterine devices, contraceptive implants, or tubal ligation as forms of modern contraceptives. A young man in the group also mentioned that he was unaware of emergency contraceptives (i.e., Postinor) prior to the discussion.

*I've learned so much (from this discussion). I know a whole lot about sex, I watch movies (American movies show the thing real) and I read it a lot. But this is the first time I heard about Postinor 2; so I've learned something*. (Male FGD)

### Source of Contraceptives

Participants described how they learned about contraceptives. Undergraduate females deferred to their friends or relatives rather than healthcare providers for advice on modern contraceptive use.

*My sister introduced me to it, that's how I got to know about postinor*.

*I actually talked to my cousin about it. She's the one that's been putting me through all this while* (Female FGD)

Healthcare providers also noted that clients often refrained from seeking professional help on the use and choice of modern contraceptives. A pharmacist expressed this concern:

*Do you know that clients believe the information they hear outside more than what they hear from medical experts?…I ask them, “have you gone to a medical practitioner to ask? So, why do you seem to have more attachment to what your friend said than getting information from a medical practitioner where you'll get the right source of information?”* (Pharmacist 2)

## Discussion

Using the theory of planned behavior as the underpinning framework, this study sought to elucidate undergraduate students' awareness of, and behaviors related to, use of contraceptives in southeast Nigeria. This study builds upon existing research by expanding on respondents' contraceptive knowledge, highlighting specific folkloric methods of contraception, and young people's preference for the withdrawal method over condoms and oral contraceptives.

With regards to behavioral beliefs related contraceptives, the folkloric, context-specific, forms of contraceptives identified in this study (i.e., laxatives, antimalarial, antibiotics, and salt solution) reflect the limited contraceptive knowledge and wrong practice of contraception among young people in the study setting. This may be interpreted as reflecting a limited understanding of sexual and reproductive health, thus, calling for a need to promote comprehensive sex education (CSE) among youths in Calabar, Nigeria. CSE includes not only the provision of accurate information on the benefits of delaying sexual debut, but also education on contraception and prevention of sexually transmitted infections (19). Such educational interventions in Nigeria are likely to be effective if designed to reflect adolescent behavioral theories of change (e.g., TpB) while taking account of the specific social and cultural contexts of the intervention setting. Strategies known to be effective among young people, including such appropriate technologies—e.g., text messaging, TikTok, snapchat, or Instagram (20)—should be piloted to assess their feasibility and effectiveness in southeast Nigeria. While action to address knowledge is important, the effect of culture and stigmatization on young people's beliefs about normative expectations of others and motivation to comply with these expectations (normative beliefs) in relation to contraceptives use must also be considered. Across many parts of Nigeria and other low-income countries there is the cultural expectation that young people should not have sex until after marriage (21). In fact, it is well-established that many family planning programs in sub-Saharan Africa do not cater to the needs of unmarried women, and hence the huge unmet need for contraception among this group (22, 23). Thus, it is often difficult for unmarried individuals (mostly females) to boldly walk into a pharmacy and request to purchase contraceptives.

Although the Nigerian educational curriculum includes elements of reproductive health, there is no evidence of CSE in its national educational curricula. An example of a sex education program in Nigeria is the Family Life and HIV/AIDS Education (FLHE), an abstinence-based intervention taught to primary school and secondary school students (24, 25). In 2003, the Nigerian Federal Ministry of Education incorporated FLHE into primary and secondary schools' syllabi. While the FHLE curriculum includes human development, HIV infection, gender-based violence, and human trafficking (24), it lacks information on contraceptive methods and their effectiveness for the cultural reasons discussed above. Also, disparity exists in the adoption and implementation of FHLE across Nigeria, and less priority is placed on university students (25). However as this study shows, many university students in Calabar lack comprehensive knowledge of sexual health.

Given the limitations of FHLE implementation in Nigeria, there is a need for targeted CSE especially among secondary and university students who may likely experience sexual debut during their training. The median age at sex debut is 17 and 22 years among women and men while the median age of first marriage is 19 and 28 years among women and men in Nigeria, respectively (6). Therefore, it is imperative to incorporate CSE into existing general educational curricula of Nigerian youths since many have intercourse at least a couple of years before they are married and may desire to delay childbearing until after marriage. CSE could also be disseminated through mass media campaigns. Mass media intervention has been used across various settings to promote contraceptive knowledge and use among youths. Contraceptive interventions via mass media campaigns result in changes in improving contraceptive attitudes, beliefs, knowledge, and use especially when mass media was combined with other components such as social marketing and interpersonal communication (26). In Nigeria, specifically, there is proof that the media has an influence on youth's sexual and reproductive health knowledge and needs (27). Adolescents also find it “cheaper” and “faster” to get health information via mass media than going to a hospital (27). Delivering CSE through educational curricula and mass media campaigns will provide Nigerian youths with tools to choose safe and effective contraceptives rather than misusing antibiotics as contraceptives.

As seen in this study and other scholarly articles, youths acquire knowledge about contraceptives from their peers, who may not possess the medically accurate knowledge of sexual health (27, 28). In their study of adolescents in Nigeria, Olumide et al. confirmed that young people refer to their peers for sexual health information even after interacting with sexual health information on mass media (27). Therefore, peer educators can refer young people to scientifically accurate and stigma free sexual health information (28). In addition, there is evidence for the effectiveness of peers in promoting sex education and safe-sex practices in other African countries. Studies from Ghana and South-Africa showed that sex education delivered by peer educators leads to improved indicators of contraceptive knowledge including a better understanding of condom use, pregnancy prevention and STI testing (29, 30). Engaging youth as peer-educators in CSE and referral to sexual and reproductive health services can help decrease the abuse of pharmaceuticals as contraceptives and promote knowledge and use of modern contraceptives when needed.

Lastly, CSE can be implemented more effectively by integrating a mass media intervention with a peer-education component. A great example of such integration is *MTV Shuga*, a peer education project, funded by the Bill & Melinda Gates Foundation (31, 32). *MTV Shuga* is aimed at improving reproductive health knowledge, attitudes, and behavior especially among women aged 15–24 (31, 32). Since its inception in 2009, the *MTV Shuga* series has been on 179 broadcasters worldwide and has reached 719 million individuals (32). The series has had a considerable impact on Nigerian youth's sexual health. First, youths who watched *MTV Shuga* relayed the messages to their peers, thus pointing to the need for working with peers in promoting safe sex (32). Second, more than one in three (35%) young Nigerians who watched the series were more likely to get tested for HIV in the past 6 months (32). Third, an evaluation of the series among Nigerian youths aged 18–25 years displayed an increase in HIV knowledge including knowledge of places to get tested, a decrease in high-risk sexual behavior, and a decrease in sexually transmitted infections (33). With such a great impact and wide reach, *MTV Shuga* can expand its scope from HIV awareness and prevention to include CSE. Emphasizing sexual education in *MTV Shuga* provides a unique avenue for correcting myths on using antimalarial, alcohol, and antibiotics as contraceptives while providing referrals to reproductive and sexual health resources.

## Limitation

While this study explores the knowledge and practice of contraceptive practices in youths, there are three limitations to this study. First, this study did not include out-of-school youths in FGDs. Discussions with out-of-school young men and women could provide more dimensions about contraceptive knowledge and practice than identified in this study. Second, patent and proprietary medicine vendors (PPMVs) were not interviewed in this study. In Nigeria, PPMVs are individuals with no formal medical training who sell pharmaceutical products on a retail basis for profit (34). Since people often seek PPMVs for pharmaceutical products and treatment of many health conditions (35), interviewing these vendors could shed light on their knowledge of sexual and reproductive health services and provide opportunities for training and referral services (36). Third, although the study was conducted in 2017, the salient results emerging from this study remain relevant.

## Recommendation and Conclusion

The limited contraceptive knowledge among university students in this study points to the dire need for CSE among university students in Calabar, Nigeria. Considering that sexual debut among many Nigerian women and men occurs at 17 and 22 years old, respectively (6), it is important to promote research and programming efforts on youth's sexual health beyond HIV awareness efforts. These efforts can be achieved through using peer-educators, wider dissemination of CSE, and provision of confidential reproductive and sexual health services. These comprehensive efforts will further mitigate the unmet need for contraceptives among Nigerian youths and lower the burden of unplanned pregnancies.

## Data Availability Statement

The raw data supporting the conclusions of this article will be made available by the authors, without undue reservation.

## Ethics Statement

The studies involving human participants were reviewed and approved by Health Research Ethics Committee, University of Calabar Teaching Hospital, Calabar, Nigeria. The patients/participants provided their written informed consent to participate in this study.

## Author Contributions

IA and JE conceptualized and designed the study. AAb, MB, and IA conducted focus group discussions and interviews. KK-L, AAb, and IA transcribed the data. NK, KK-L, and IA analyzed the data. CA, EA, IA, AAs, and JE developed the manuscript and revised the manuscript. All authors read and approved the final manuscript.

## Conflict of Interest

The authors declare that the research was conducted in the absence of any commercial or financial relationships that could be construed as a potential conflict of interest.
